# Book Reviews

**Published:** 1897-08

**Authors:** 


Reference-Book of Practical Therapeutics. By various authors. Edited by Frank P. Foster, M. D., Editor of the New York Medical Journal and of Foster's Encyclopedic Medical Dictionary. In two large octavo volumes. Volumes I. and II. Pp. vii.—632 and 618. New York: D. Appleton & Co. 1897.

This work is encyclopedic in character and the signature of each contributor is appended to his offering. It is not intended to supplant the text-books on materia medica, pharmacy or therapeutics, but its object is rather to supplement these by placing in the hand of the practising physician a list of his therapeutic armamentarium alphabetically arranged. We use the word list advisedly; we do not mean to be understood thereby, however, that drugs, medicines and therapeutic appliances are only mentioned in this treatise by name. Far from it. In most instances chemical formulæ or botanical histories are given, the nature of the products described, their therapeutic application detailed, and in case of poisons, antidotes and curative management are set forth.

To do all this requires two imperial double-columned octavo volumes of 650 pages each, which indicates the magnitude of the work. Moreover, it is printed in fine type, yet with such a clear face that it does not tax the eye even of advanced age to find and read all the practical information necessary on a given subject. But, withal, the editor has mastered the task of presenting to us in most approved form the essential information of all remedial agents that are in vogue. He has drawn upon current periodic literature extensively wherever necessary to elaborate his subject, which adds very much to its interest as well as practical importance.

It is not a treatise for the student, but it is essentially a work for the practical and practising physician—for the family doctor, the consultant and the specialist. All these may daily consult these volumes with advantage. The specialist must always know the action of remedies and those best adapted to his patient; for even if he does not prescribe them he must not be ignorant on the subject nor fail to appreciate the proper work of him whose duty it is to administer or employ internal remedies.

We are unhesitatingly of the opinion that this treatise will easily become the most popular reference-book in a physician's library, and that to be without it is to miss the most practical and useful work on therapeutics extant.

Transactions of the American Association of Obstetricians and Gynecologists. Volume IX. for the year 1896. Standard 8vo, pp. viii.—456. Edited by the secretary, William Warren Potter, M. D. Philadelphia: William J. Dornan. 1897.

In this volume is printed the proceedings, papers and discussions of the Richmond meeting, September 22–24, 1896, under the presidency of Dr. Joseph Price. It is a picture of clinical work in the consulting room, at the bedside and at the operating table for a year, by a group of progressive, earnest men, who succeed in making the most interesting volume of society transactions that we have seen.

The book is well printed, profusely illustrated and substantially as well as handsomely bound, and ought to be in possession of every abdominal surgeon, obstetrician and gynecologist in America.

---

Atlas and Essentials of Gynecology. By Dr. Oscar Schaeffer, University of Heidelberg. Sixty-four full-page chromo-lithographic plates, comprising 159 figures; with descriptive text facing the plates and over 300 pages of introduction and treatise, illustrated with wood-engravings. (Wood's Medical Hand Atlases, Volume V.)

In this Atlas the author has combined schematic drawings with those taken from nature and has succeeded in plainly emphasising many variations from the normal that many other works leave obscure or only partially understood. In doing this he has drawn largely upon material that he obtained when assistant at the gynecological clinic at Munich. The work is divided into two parts—first, plates and their explanation; then text with wood-cut illustrations.

Schaeffer has succeeded in presenting a volume of great beauty that will materially aid in the elucidation of many gynecological subjects and one that will readily become a work of great supplemental force in the hands of the gynecologist.

Its moderate price will easily commend it to the general practitioner, who must of necessity have an intelligent knowledge of ordinary gynecologic subjects.

Warner's Pocket Medical Dictionary of Today. Comprising Pronunciation and Definition of 10,000 Essential Words and Terms used in Medicine and Associated Sciences. By William R. Warner. Philadelphia: William R. Warner & Co. 1897.

Small dictionaries are often very convenient and this one, giving 10,000 words, most of which are in everyday use, is one of the best of its kind. There are numerous minor errors, many of which are attributable to faulty proof-reading and some to condensed definitions. Nevertheless, it is a book that possesses merit; its letter-face is plain, diphthongs have been omitted wherever practicable and it admirably fills the place for which it is intended—namely, the pocket.

